# First report of *Taenia arctos* (Cestoda: Taeniidae) from brown bears (*Ursus arctos*) in Romania

**DOI:** 10.1186/s13071-025-07221-z

**Published:** 2026-01-05

**Authors:** Maria Monica Florina Moraru, Andrei-Daniel Mihalca, Ana-Maria Marin, Dan-Cornel Popovici, Azzurra Santoro, Sorin Morariu, Ioana Bianca Mitrea, Narcisa Mederle

**Affiliations:** 1 Department of Parasitology and Parasitic Diseases, Faculty of Veterinary Medicine, University of Life Sciences “King Mihai I” From Timisoara, Calea Aradului, No. 119, 300645 Timisoara, Romania; 2 Department of Parasitology and Parasitic Diseases, Faculty of Veterinary Medicine, University of Agricultural Sciences and Veterinary Medicine of Cluj-Napoca, Calea Mănăştur, No. 3-5, 400372 Cluj-Napoca-Napoca, Romania; 3 Forestry Faculty, Transilvania University Brasov, Șirul Ludwig Van Beethoven, no.1, 500123 Brasov, Romania; 4 WHO Collaborating Centre for the Epidemiology, Detection and Control of Cystic and Alveolar Echinococcosis (One Health, Department of Infectious Diseases, Istituto Superiore Di Sanità, Rome, Italy; 5 European Union Reference Laboratory for Parasites (EURL-P), Department of Infectious Diseases, Istituto Superiore Di Sanità, Viale Regina Elena 299, 00161 Rome, Italy

**Keywords:** *Ursus arctos*, *Taenia arctos*, Romania, Molecular identification

## Abstract

**Background:**

The brown bear (*Ursus arctos*) is an apex predator with significant ecological importance and serves as a valuable indicator species for monitoring parasitic burdens in forest ecosystems. Owing to its complex ecology and varied diet, this species may play a key role in the life cycle of numerous pathogens, including cestodes of the genus *Taenia*, such as the recently described *Taenia arctos*. Among the potential intermediate hosts for *T. arctos* is the moose (*Alces alces*), a species sporadically present in northern Romania, where its habitat may overlap with that of *U. arctos*. In this context, the present study aimed to identify and molecularly characterize cestodes isolated from the small intestines of *U. arctos*.

**Methods:**

Between May 2022 and December 2024, small intestines from 91 *U. arctos* individuals were collected across 16 counties in Romania. Biological samples were preserved by freezing and analyzed using both classical methods (macroscopic and microscopic examination) and molecular biology techniques (through amplification and sequencing of mitochondrial gene fragments, *cox1* and 12S rRNA).

**Results:**

Out of the 91 samples analyzed, only 1 specimen tested positive for an adult cestode (prevalence 1.1%). Genetic analysis confirmed its identification as *T. arctos*, a species not previously reported in Romania.

**Conclusions:**

This study represents the first molecular identification of *T. arctos* in *U. arctos* in southeastern Europe, thereby extending the known geographic range of the parasite. The findings may indicate that trophic interactions compatible with the life cycle of *T. arctos* occur in this area, although the evidence is limited to a single detection.

**Graphical Abstract:**

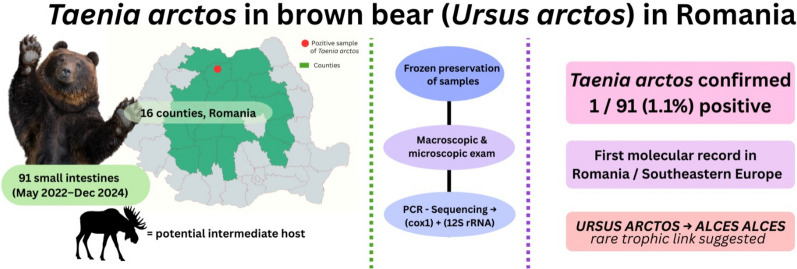

## Background

Parasite diversity can serve as a bioindicator of ecosystem health and, implicitly, of the evolutionary mechanisms and conservation of endangered species [1–3]. Parasites with more complex, heteroxenous life cycles are more intimately associated with ecosystem functions and interspecific interactions, mainly those transmitted via predatory behavior, such as tapeworms. Among these, species of the genus *Taenia* (Taeniidae) are characterized by the development of the adult stage in the small intestine of carnivorous or scavenger mammals, while the larval stages develop in the tissues and organs of various prey mammals, which act as intermediate hosts. *Taenia arctos* has been described in various bear species (Ursidae), with larval cestodes identified in various species of ungulates [4–7].

*Taenia arctos* shows an Holarctic distribution, identified in Finland [4] and Canada [5, 6], and displays host specificity for ursids (*Ursus arctos arctos*, *U. a. horribilis*, and *U. americanus*) as definitive hosts, and cervids (*Alces alces*, and *Alces americanus*) as intermediate hosts. The life cycle involves the development of *Cysticercus*-type larvae in the muscles of intermediate hosts, followed by the development of adults in the small intestine of bears after ingestion of infected tissues [4–6].

As a member of the large carnivores’ group, the brown bear, *U. arctos* is an apex predator that has inhabited Romania’s forest ecosystems since ancient times [8]. The species exhibits remarkable adaptability and ecological plasticity, easily shifting between the roles of apex predator, scavenger, and frugivore. Its ecology, wide distribution, and frequent interactions with humans and domestic animals position it as suitable biological model for monitoring parasites in wild populations [3]. Romania hosts over 35% of the European brown bear population [9]. The species maintains consistent patterns of daily activity and seasonal migration, depending on climatic conditions and food availability [10], during which it demonstrates its adaptive capabilities as an omnivore and/or apex predator [11, 12].

In Europe, *U. arctos* has been reported to be infected with several groups of intestinal parasites [13–15], including cestodes such as *Diphyllobothrium* [16] and *Anoplocephala* [17]. Despite the abundant bear population in Romania, studies on the parasites of *U. arctos* are limited to occasional reports. Necropsy and coproscopy performed in *U. arctos* collected in the Transylvania region revealed six species of nematodes, one protozoan, an unidentified species of Taeniidae, and the trematode *Dicrocoelium dendriticum* [18].

In the context of scarce knowledge on the real distribution and host range of *T. arctos*, the aim of the present study was to provide new data regarding its geographical distribution in Romania, the country with the highest brown bear population in Europe.

## Methods

**Fig. 1:**
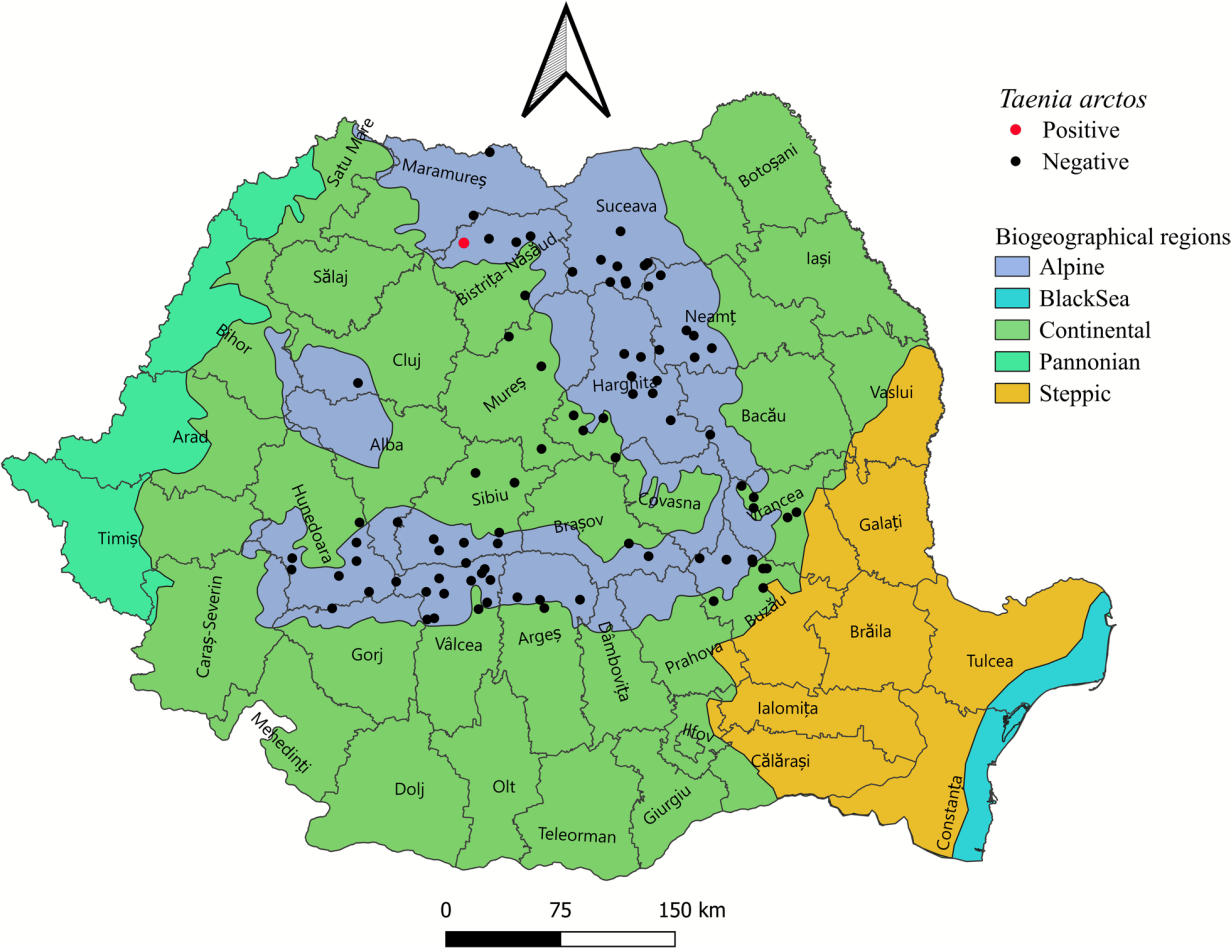
Map showing the geographical locations (counties) of the tested animals and the *T. arctos* positive animal

The study was conducted between May 2022 and December 2024 on a total of 91 hunted *U. arctos* individuals (73 males and 18 females) originating from hunting grounds across 16 counties in Romania (Fig. 1). The samples analyzed in the present study were obtained exclusively from *U. arctos* individuals legally hunted in accordance with national legislation. For each specimen, an official derogation was issued by the Ministry of Environment, Waters and Forests: in 2022 (Ministerial Order No. 723/2022), in 2023 (Ministerial Order No. 2183/2023) [19], and in 2024 (Law No. 242/2024) [20]. After collection, all internal organs were frozen at −20 °C and transported to the Department of Parasitology and Parasitic Diseases, Faculty of Veterinary Medicine, University of Life Sciences “King Mihai I” from Timişoara. Following biosecurity protocols being implemented in accordance with international guidelines issued by the World Organisation for Animal Health (WOAH) [21] and the European Food Safety Authority (EFSA) [22]; all 91 samples were kept at −80 °C for 48 h prior to processing, to inactivate potential zoonotic agents. Subsequently, the small intestine of each specimen was longitudinally sectioned, and the intestinal content was examined macroscopically [23]. All cestode-like helminths were collected in Petri dishes in physiological saline and examined microscopically for morphological identification. However, due to deep freezing, which affected the integrity of the helminths, standard morphological identification was not possible. Consequently, a simplified method was employed and proglottids from the terminal portion of the cestode were collected, compressed between two microscope slides, and examined microscopically [23]. The remaining tapeworms were stored in 96% ethanol for molecular characterization.

Genomic DNA was isolated from proglottids using the DNeasy Blood & Tissue Kit (Qiagen, Valencia, CA, USA) according to the manufacturer’s instructions. A negative control consisting of nuclease-free water was incorporated in each processing session to ensure the absence of contamination. The extracted DNA was subsequently stored at −20 °C until further analysis. A fragment of the mitochondrial cytochrome oxidase I gene (*cox1*) was amplified using primers EgCOI 1 and EgCOI 2, originally designed by Bowles and colleagues [24] and later modified [25]. The PCR reaction was performed in a final volume of 30 μL and comprised 2 μL of DNA template, 15 μL of PCR Master Mix HotStart (Qiagen GmbH, Hilden, Germany), 0.5 μM of each primer (forward and reverse), and 10 μL nuclease-free water. The cycling conditions were as follows: an initial denaturation step at 95 °C for 15 min; 38 cycles of: denaturation 94 °C for 30 s, annealing 55 °C for 30 s, and elongation 72 °C for 30 s; and a final extension step of 5 min at 72 °C. A fragment of the 12S rRNA gene was amplified using the P60 and P375 primers [26]. The PCR reaction was performed in a final volume of 50 μL, and consisted of 5 μL of DNA template, 25 μL of PCR Master Mix HotStart (Qiagen GmbH, Hilden, Germany), 0.5 μM of each primer (forward and reverse), and 15 μL nuclease-free water. The cycling conditions were as follows: an initial denaturation step at 95 °C for 15 min; 40 cycles of denaturation 93 °C for 60 s, annealing 55 °C for 90 s, and elongation 72 °C for 2 min, and a final extension step of 5 min at 72 °C. A negative control (in which nuclease-free water replaced the DNA template) was included. PCR products were visualized by capillary gel electrophoresis (Qiaxcel, Qiagen GmbH, Hilden, Germany). After confirmation of the capillary gel electrophoresis, the PCR products were sent to GENEWIZ (Leipzig, Germany) for purification and Sanger sequencing.

Phylogenetic analysis was reconstructed with maximum likelihood (ML) inference using a multiple *cox1* alignment, including the sequences obtained in this study as well as sequences available for representative *Taenia* species in GenBank. The Hasegawa–Kishino–Yano substitution model was chosen according to the model selection function of MEGA12 software [27]. In total, 1000 bootstrap replicates were performed to estimate branch robustness.

## Results

Out of the 91 *U. arctos* specimens examined, only 1 individual, originating from Târlișua (Bistrița-Năsăud County), was found to be infected with a single cestode located in the anterior half of the small intestine. Morphological identification to the genus or species level was not possible owing to the degraded condition of the cestode. However, the microscopic examination of the compressed proglottid allowed for the identification of eggs characteristic of the family Taeniidae.

**Fig. 2:**
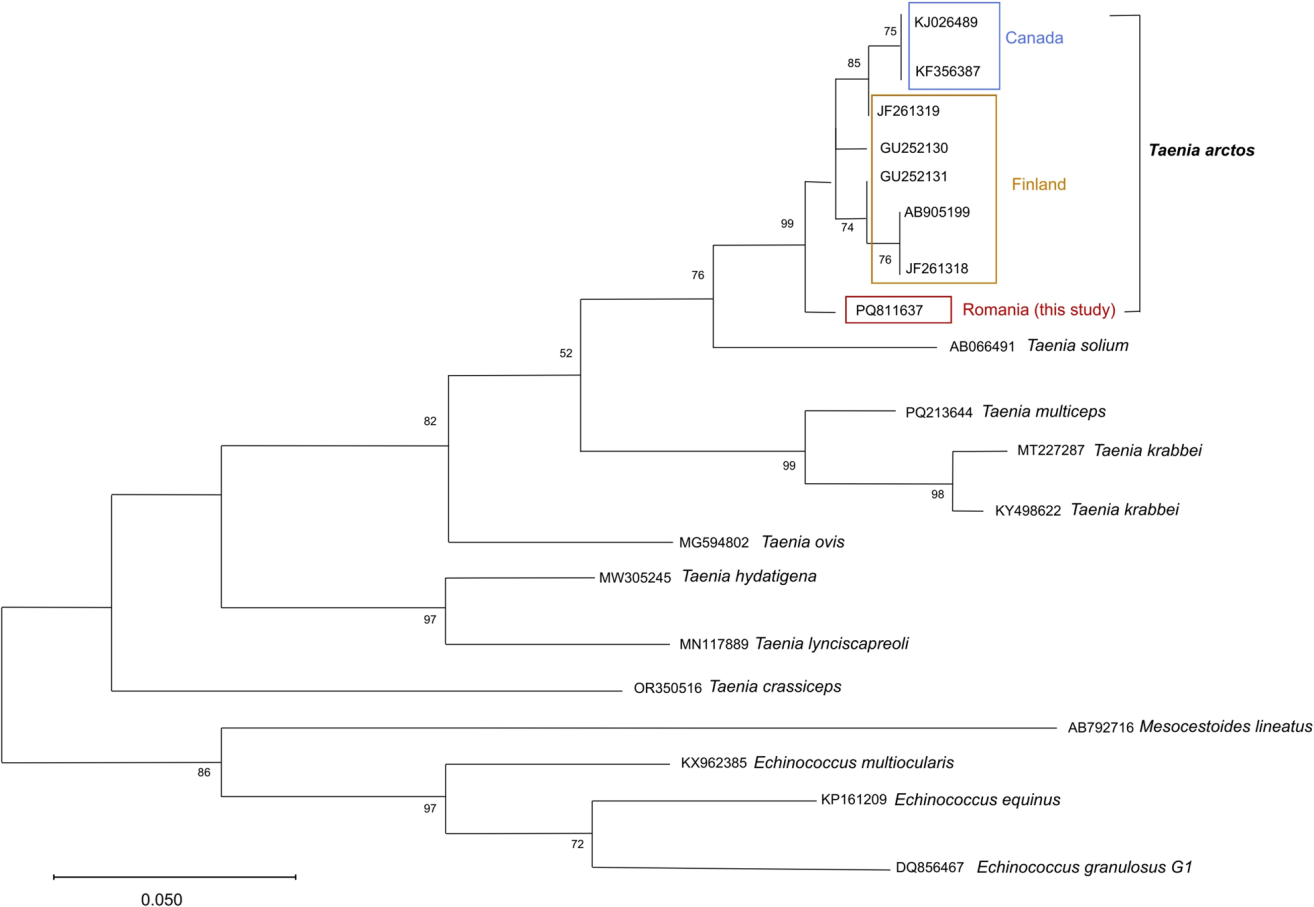
Phylogenetic tree inferred by the maximum likelihood method and Hasegawa–Kishino–Yano model using nucleotide sequences (396 sites) of the *cox1* gene. GenBank accession numbers are shown. *cox1 T. arctos* sequence code from this study is red-squared. Branch quality was determined by bootstrap analysis with 1000 replicates. Bootstrap percentages are shown when the value exceeds 50%

The cestode was molecularly identified as *T. arctos* on the basis of sequence similarity of the *cox1* (419 base pairs) and 12S (342 base pairs) mitochondrial gene regions. For *cox1* (PQ811637) and the 12S (PQ811837), the BLAST analysis showed 99.29% and 99.78% identity, respectively, with the AB905199 reference mitochondrial genome deposited in GenBank [28]. The phylogenetic analysis confirmed the species assignment, although it did not infer a specific evolutionary proximity with available *Taenia arctos* sequences, which originated from Finland and Canada, respectively (Fig. 2).

## Discussion

This study presents the first molecularly confirmed report of *T. arctos* in Romania, and to our knowledge, the first in Eastern Europe. *T. arctos* was described in brown bears (*Ursus arctos*) as definitive hosts and Eurasian moose as intermediate hosts in Finland [4]. Subsequently, *T. arctos* has been reported in other ursids such as grizzly (*U. arctos horribilis*) and black bears (*U. americanus*), from Alberta and British Columbia, Canada [5, 6]. The identification of *T. arctos* in the Carpathian population of *Ursus arctos* extends the confirmed distribution of the species. However, given the single detection, any inference regarding the true prevalence or broader distribution should be made cautiously.

The parasite’s life cycle involves definitive hosts in the family Ursidae and intermediate hosts in the genus *Alces*. Larval cysticerci have been found in the skeletal muscles of Eurasian elk (*Alces alces*) and North American moose (*A. americanus*) [4, 29]. In our study, the presence of an adult *T. arctos* in a Romanian brown bear raises questions about the current or historical presence of suitable intermediate hosts. Moose are rarely observed in Romania and are not known to maintain established populations. *Alces alces* has a sporadic and occasional presence in Romania’s fauna [30], being mainly associated with boreal forest habitats characterized by a mosaic structure of pastures and vegetation [31, 32].

Therefore, the detection of *T. arctos* in this region may suggest one of the following: (*i*) spillover from transient moose, (*ii*) occasional long-distance infections, or, more likely, (*iii*) the existence of other cervid species acting as intermediate hosts. Although only *Alces* spp. have been confirmed to harbor larvae of *T. arctos*, the parasite may exhibit broader intermediate host plasticity than currently recognized. Red deer (*Cervus elaphus*), roe deer (*Capreolus capreolus*), and fallow deer (*Dama dama*), all of which are common in Romanian forests, could potentially serve as intermediate hosts but have not been investigated using molecular tools for this parasite. Given the morphological similarity between *T. arctos* and *T. krabbei* at both larval and adult stages, historical records [33, 34] may have potentially misclassified *T. arctos* as *T. krabbei*, particularly in regions where both parasites might co-exist [4, 5].

The low prevalence observed in this study (1.1%) aligns with reports from other regions: 1.9% in Finnish brown bears [4] and 7.7% in Canadian bears [6]. These values suggest that *T. arctos* is generally rare, or that its detection is underestimated owing to the limitations of morphology-based diagnostics. Indeed, *T. arctos* was previously misidentified in a Greenland muskox as *T. ovis krabbei* until molecular evidence revealed its true identity [5, 35].

Molecular characterization has been critical in differentiating *T. arctos* from other taeniids. The mitochondrial *cox1* and 12S sequences used in this study shift the southern edge limit of *T. arctos* to northern Romania, supporting a broader Holarctic distribution than previously inferred. The low genetic divergence between Palearctic and Nearctic haplotypes suggests a relatively recent common ancestry and possible post-glacial range expansion, facilitated by host movements across the Bering Land Bridge during the Pleistocene [5].

## Conclusions

Our findings expand the known range of *T. arctos* to Eastern Europe and highlight the need for broader surveillance of both definitive and potential intermediate hosts across Europe. The possibility that other cervids serve as intermediate hosts deserves urgent investigation, using molecular methods to re-examine taeniid larvae from wild ungulates across the region. These efforts will be essential to clarify the ecology, host specificity, and evolutionary history of this elusive parasite. However, the conclusions are based on a single infected individual, and therefore prevalence estimates, host associations, and ecological interpretations must be considered preliminary.

## Data Availability

Data supporting the main conclusions of this study are included in the manuscript.

## References

[1] Kutz SJ, Ducrocq J, Verocai GG, Hoar BM, Colwell DD, Beckmen KB, et al. Parasites in ungulates of Arctic North America and Greenland: a view of contemporary diversity, ecology, and impact in a world under change. Adv Parasitol. 2012;79:99–252. 10.1016/B978-0-12-398457-9.00002-0. 22726643 10.1016/B978-0-12-398457-9.00002-0

[2] Hwang MH, Chin TW, Yu PH. Endoparasites of Formosan black bears during acorn season. J Wildl Dis. 2021;57:345–56. 10.7589/JWD-D-20-00067. 33822155 10.7589/JWD-D-20-00067

[3] Roncancio-Duque N. Parasites in bears (Ursidae): sampling gaps in the spectacled bear (*Tremarctos ornatus*). Rev Med Vet. 2024;49:1–10. 10.19052/mv.vol1.iss49.2.

[4] Haukisalmi V, Lavikainen A, Laaksonen S, Meri S. *Taenia arctos* n. sp. from brown bear and moose hosts. Syst Parasitol. 2011;80:217–30. 10.1007/s11230-011-9324-9. 22002024 10.1007/s11230-011-9324-9

[5] Catalano S, Lejeune M, Verocai GG, Duignan PJ. First report of *Taenia arctos* (Cestoda: Taeniidae) from grizzly (*Ursus arctos horribilis*) and black bears (*Ursus americanus*) in North America. Parasitol Int. 2014;63:389–91. 10.1016/j.parint.2013.12.012. 24382413 10.1016/j.parint.2013.12.012

[6] Catalano S, Lejeune M, Tizzani P, Verocai GG, Schwantje H, Nelson C, et al. Helminths of grizzly bears (*Ursus arctos*) and American black bears (*Ursus americanus*) in Alberta and British Columbia, Canada. Can J Zool. 2015;93:765–72. 10.1139/cjz-2015-0063.

[7] Haukisalmi V. Checklist of tapeworms (Platyhelminthes, Cestoda) of vertebrates in Finland. ZooKeys. 2015;533:1–61. 10.3897/zookeys.533.6538. 10.3897/zookeys.533.6538 PMC4669923 26668540

[8] Micu I. Ursul brun: aspecte eco-etologice. Bucharest: ed. Ceres; 1998.

[9] Popa M, Jurj R, Sârbu G, Ionescu G, Fedorca A, Ionescu O. Home range, daily and seasonal activity of brown bear (*Ursus arctos*) in South-Eastern Carpathians – a GPS/GSM telemetry study. In: Proc Biennal Int Symp Forest Sustain Dev. 8th ed. 25–27 October Brașov, Romania. 2018.

[10] De Angelis D, Huber D, Reljic S, Ciucci P, Kusak J. Factors affecting the home range of Dinaric-Pindos brown bears. J Mammal. 2021;102:481–93. 10.1093/jmammal/gyab018.

[11] Dahle B, Wallin K, Cederlund G, Persson IL, Selvaag LS, Swenson JE. Predation on adult moose *Alces alces* by European brown bears *Ursus arctos*. Wildlife Biol. 2013;19:165–9. 10.2981/10-113.

[12] Bogdanović N, Hertel AG, Zedrosser A, Paunović M, Plećaš M, Ćirović D. Seasonal and diel movement patterns of brown bears in southeastern Europe. Ecol Evol. 2021;11:15972–83. 10.1002/ece3.8267. 34824804 10.1002/ece3.8267 PMC8601923

[13] Aghazadeh M, Elson-Riggins J, Reljić S, De Ambrogi M, Huber Đ, Majnarić D, et al. Gastrointestinal parasites and the first report of *Giardia* spp. in a wild population of European brown bears (*Ursus arctos*) in Croatia. Vet Arhiv. 2015;85:201–10.

[14] Molnár L, Königová A, Major P, Vasilková Z, Tomková M, Várady M. Seasonal pattern of prevalence and excretion of *Baylisascaris transfuga* in the brown bear (*Ursus arctos*). Animals. 2020;10:2428. 10.3390/ani10122428. 33353114 10.3390/ani10122428 PMC7767011

[15] Costa H, Hartasánchez R, Santos AR, Camarão A, Cruz L, Nascimento M, et al. Preliminary findings on the gastrointestinal parasites of the brown bear (*Ursus arctos*) in the Cantabrian Mountains, Spain. Vet Parasitol Reg Stud Rep. 2022;28:100681. 10.1016/j.vprsr.2021.100681. 10.1016/j.vprsr.2021.100681 35115125

[16] Yamasaki Y, Muto M, Yamada M, Arizono N, Rausch RL. Validity of the bear tapeworm *Diphyllobothrium ursi* (Cestoda: Diphyllobothriidae) based on morphological and molecular markers. J Parasitol. 2012;98:1243–7. 10.1645/GE-3063.1. 22663179 10.1645/GE-3063.1

[17] Bugmyrin SV, Tirronen KF, Panchenko DV, Kopatz A, Hagen SB, Eiken HG, et al. Helminths of brown bears (*Ursus arctos*) in the Kola Peninsula. Parasitol Res. 2017;116:1755–60. 10.1007/s00436-017-5456-4. 28484854 10.1007/s00436-017-5456-4

[18] Borka-Vitális L, Domokos C, Földvári G, Majoros G. Endoparasites of brown bears in eastern Transylvania, Romania. Ursus. 2017;28:20–30. 10.2192/URSU-D-16-00015.1.

[19] Ministerul Mediului, Apelor și Pădurilor. Ordinul nr. 2183/2023 pentru aprobarea nivelului de intervenție și de prevenție în cazul speciei urs brun (Ursus arctos), în interesul sănătății și securității populației și în scopul prevenirii unor daune. Monitorul Oficial al României, Partea I, nr. 764, 23 august 2023. Disponibil online: https://lege5.ro/Document/geztsnjtgu2dg Accessed 15 may 2025.

[20] Ministerul Mediului, Apelor și Pădurilor. Legea nr. 242/2024 pentru completarea art. 1 din Legea vânătorii și a protecției fondului cinegetic nr. 407/2006, precum și pentru modificarea și completarea Ordonanței de urgență a Guvernului nr. 81/2021 privind aprobarea metodelor de intervenție imediată pentru prevenirea și combaterea atacurilor exemplarelor de urs brun asupra persoanelor și bunurilor acestora, precum și pentru modificarea și completarea unor acte normative. Monitorul Oficial al României, Partea I, nr. 720, 23 iulie 2024. Disponibil online: https://lege5.ro/Document/ge2tgojwgy3di Accessed 15 may 2025.

[21] World Organisation for Animal Health (WOAH). The WOAH register. https://www.woah.org/en/home/. Accessed 13 May 2025.

[22] European Food Safety Authority (EFSA). The EFSA register. https://www.efsa.europa.eu/en. Accessed 13 May 2025.

[23] Zajac AM, Conboy GA, Little SE, Reichard MV. Veterinary Clinical Parasitology. Hoboken: Wiley-Blackwell; 2021.

[24] Bowles J, Blair D, McManus DP. Genetic variants within the genus *Echinococcus* identified by mitochondrial sequencing. Mol Biochem Parasitol. 1992;54:165–74. 10.1016/0166-6851(92)90109-W. 1435857 10.1016/0166-6851(92)90109-w

[25] Bart JM, Morariu S, Knapp J, Ilie MS, Pitulescu M, Anghel A, et al. Genetic typing of *Echinococcus granulosus* in Romania. Parasitol Res. 2006;98:130–7. 10.1007/s00436-005-0015-9. 16328370 10.1007/s00436-005-0015-9

[26] von Nickisch-Rosenegk M, Lucius R, Loos-Frank B. Contributions to the phylogeny of the Cyclophyllidea (Cestoda) inferred from mitochondrial 12S rDNA. J Mol Evol. 1999;48:586–96. 10.1007/PL00006501. 10198124 10.1007/pl00006501

[27] Kumar S, Stecher G, Suleski M, Sanderford M, Sharma S, Tamura K. MEGA12: molecular evolutionary genetic analysis version 12 for adaptive and green computing. Mol Biol Evol. 2024;41:msae263. 10.1093/molbev/msae263. 39708372 10.1093/molbev/msae263 PMC11683415

[28] National Institutes of Health (HIF). The HIF register. https://www.ncbi.nlm.nih.gov/nucleotide/AB905199.1?report=genbank&log$=nuclalign&blast_rank=2&RID=3XDPSHHK013 Accessed 2 May 2025.

[29] Lavikainen A, Laaksonen S, Beckmen K, Oksanen A, Isomursu M, Meri S. Molecular identification of *Taenia* spp. in wolves, brown bears, and cervids from North Europe and Alaska. Parasitol Int. 2011;60:289–95. 10.1016/j.parint.2011.04.004. 21571090 10.1016/j.parint.2011.04.004

[30] Belova O, Sezikas K. Dynamics and sustainable use of moose (*Alces alces*) populations. Balt For. 2017;23:711–23.

[31] Niedziałkowska M, Hundertmark KJ, Jędrzejewska B, Niedziałkowski K, Sidorovich VE, Górny M, et al. Spatial structure in European moose (*Alces alces*): genetic data reveal a complex population history. J Biogeogr. 2014;41:2173–84. 10.1111/jbi.12362.

[32] Romportl D, Bláhová A, Andreas M, Chumanová E, Anděra M, Červený J. Current distribution and habitat preferences of red deer and Eurasian elk in the Czech Republic. Eur J Environ Sci. 2017;7:50–62. 10.14712/23361964.2017.5.

[33] Frechette JL, Rau ME. Helminths of the black bear in Quebec. J Wildl Dis. 1977;13:432–4. 10.7589/0090-3558-13.4.432. 24228968 10.7589/0090-3558-13.4.432

[34] Duffy MS, Greaves TA, Burt MD. Helminths of the black bear, *Ursus americanus*, in New Brunswick. J Parasitol. 1994;80:478–80. 10.2307/3283422. 8195953

[35] Raundrup K, Al-Sabi MNS, Kapel CMO. First record of *Taenia ovis krabbei* muscle cysts in muskoxen from Greenland. Vet Parasitol. 2012;184:356–8. 10.1016/j.vetpar.2011.09.010. 21955737 10.1016/j.vetpar.2011.09.010

